# Trifloroside Induces Bioactive Effects on Differentiation, Adhesion, Migration, and Mineralization in Pre-Osteoblast MC3T3E-1 Cells

**DOI:** 10.3390/cells11233887

**Published:** 2022-12-01

**Authors:** Hyung-Mun Yun, Bomi Kim, Ji Eun Park, Kyung-Ran Park

**Affiliations:** 1 Department of Oral and Maxillofacial Pathology, School of Dentistry, Kyung Hee University, Seoul 02447, Republic of Korea; 2 National Development Institute for Korean Medicine, Gyeongsan 38540, Republic of Korea; 3 Gwangju Center, Korea Basic Science Institute (KBSI), Gwangju 61751, Republic of Korea

**Keywords:** autophagy, necroptosis, Gentianae Scabrae Radix, osteoblast differentiation, RUNX2, trifluoroside

## Abstract

Gentianae Scabrae Radix is used in traditional medicine and is known to possess bioactive compounds, including secoiridoid glycosides, flavonoids, lignans, and triterpenes. Trifloroside (TriFs) is a secoiridoid glycoside known for its antioxidant activity; however, its other effects have not been studied. In the present study, we investigated the biological effects of TriFs isolated from the roots of Gentianae Scabrae Radix using pre-osteoblast MC3T3E-1 cells. No cellular toxicity was observed with 1 μM TriFs, whereas 5–100 μM TriFs showed a gradual increase in cell viability. Alkaline phosphatase staining and microscopic observations revealed that 1–10 μM TriFs stimulated osteogenic activity during early osteoblast differentiation. Trifloroside also increased mineral apposition during osteoblast maturation. Biochemical analyses revealed that TriFs promoted nuclear RUNX2 expression and localization by stimulating the major osteogenic BMP2-Smad1/5/8-RUNX2 pathway. Trifloroside also increased p-GSK3β, β-catenin, p-JNK, and p-p38, but not Wnt3a, p-AKT, and p-ERK. Moreover, TriFs increased the MMP13 levels and promoted cell migration and adhesion. In contrast, TriFs-induced osteoblast differentiation and maturation had negligible effects on autophagy and necrosis. Our findings suggest that TriFs induces osteogenic effects through differentiation, adhesion, migration, and mineral apposition. Therefore, TriFs is suggested as a potential drug target in osteoblast-mediated bone diseases.

## 1. Introduction

Bone metabolism and homeostasis are tightly maintained through physiological responses associated with mesenchymal stem cell-derived osteoblast differentiation and maturation [1]. Osteoblast differentiation is induced and controlled through complex signaling pathways, primarily Wnt3a- and bone morphogenetic protein 2 (BMP2)-mediated signaling molecules, as well as mitogen-activated protein kinases (MAPKs) and AKT, which induce the expression of Runt-related transcription factor 2 (RUNX2) and regulate the transcriptional activity in osteoblast differentiation [2,3,4]. Consequently, osteoblast differentiation and maturation produce osteoids through synthesis and secretion to construct the bone matrix, which then leads to mineralization of the bone matrix filled around collagen rope containing dense and irregular crystals of hydroxyapatite that bestow rigidity to the bone [5,6]. In contrast, the dysregulation and impairment of osteoblast differentiation and maturation are mainly responsible for the pathogenesis of diseases including osteoporosis-induced bone fractures and periodontitis-induced alveolar bone loss [7,8].

Plant-derived compounds are used in traditional medicines and are an important source for the identification of new drugs for various diseases, including bone diseases [9]. Extracts of Gentianae Scabrae Radix used in traditional medicine contain mainly secoiridoid glycosides as well as flavonoids, lignans, and triterpenes, which have multiple effects, including anti-inflammatory activities [10,11,12]. A secoiridoid glycoside, trifloroside (TriFs), has not been reported previously for its biological effects, except for its antioxidant activity in a hippocampal neuronal cell line [13].

In the present study, TriFs was purified from the roots of Gentianae Scabrae Radix and its osteogenic effects and influence on the mechanisms of osteoblast differentiation and maturation were investigated in pre-osteoblasts.

## 2. Results

### 2.1. Isolation of TriFs from Gentianae Scabrae Radix and Its Effect on Cellular Toxicity

**Figure 1:**
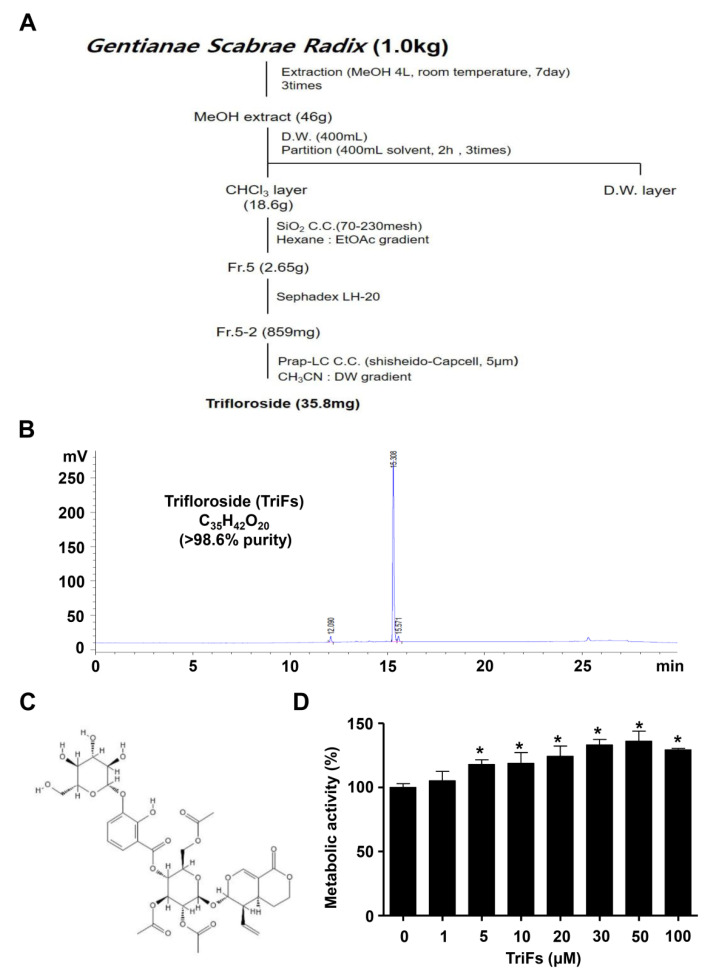
Purification of trifloroside (TriFs) from Gentianae Scabrae Radix roots and its effect on MTT assay. (**A**) Procedure for extracting TriFs from the roots of Gentianae Scabrae Radix. (**B**,**C**) high-performance liquid chromatography (HPLC) (**B**) and structure (**C**) of TriFs (C_35_H_42_O_20_, >98.6% purity). (**D**) Cell metabolic activity was determined using an MTT assay after pre-osteoblasts were treated with TriFs at doses of 1–100 μM for 24 h. *, *p* < 0.05 indicates significant differences when compared to the control. Data are presented as mean ± standard error of the mean (S.E.M.). Data shown are from three independent trials.

Gentianae Scabrae Radix (1 kg) was extracted with MeOH (4 L, three times) at room temperature for 7 days. MeOH extracts (46 g) were suspended in distilled water and then solvent partitioned with *n*-hexane and chloroform (CHCl_3_). The CHCl_3_ soluble fraction (18.6 g) was subjected to silica gel column chromatography and eluted with a gradient of *n*-hexane-EtOAc (10:0 to 0:10, *v*/*v*). Fraction 5 (2.65 g) was further purified using Sephadex LH-20 column chromatography and eluted with a MeOH-H_2_O gradient solvent system (60:40 to 0:100, *v*/*v*) to obtain compounds (859 mg). Fraction 5-2 was applied to reversed-phase high-performance liquid chromatography with a gradient system of acetonitrile-H_2_O (2:8 → 10:0) to obtain compound 1 (35.8 mg). The isolation procedure is shown in Figure 1A. The structure of compound 1 was identified as TriFs by comparison of the nuclear magnetic resonance (NMR) data with reports in previous literature [14]. The ^13^C-NMR values were (63 MHz, CD_3_OD) δ 98.6 (C-2), 153.3 (C-3), 106.7 (C-4), 28.7 (C-5), 25.7 (C-6), 70.9 (C-7), 132.7 (C-8), 43.4 (C-9), 121.3 (C-10), 167.9 (C-11), 97.6 (C-1′), 72.9 (C-2′), 72.3 (C-3′), 71.3 (C-4′), 73.3 (C-5′), 114.6 (C-1″), 152.7 (C-2″), 147.3 (C-3″), 125.0 (C-4″), 120.2 (C-5″), 124.3 (C-6″) 103.4 (C-1‴), 74.8 (C-2‴), 77.7 (C-3‴), 69.9 (C-4‴), 78.3 (C-5‴), 62.5 (C-6‴), 172.1, 171.3, 171.0, 20.6, 20.5, 20.4 (3 × OAc) (Supplementary Figure S1A). Values for ^1^H-NMR were (250 MHz, CD_3_OD) δ 7.58 (1H, d, *J* = 2.45 Hz, H-3), 7.47 (1H, dd, *J* = 1.45, 17.68 Hz, H-4″), 7.43 (1H, dd, *J* = 1.45, 17.68, H-6′), 6.87 (1H, t, H-5”), 2.01 (3H, s, AcO), 1.96 (3H, s, AcO), 1.93 (3H, s, AcO) (Supplementary Figure S1B). The high-performance liquid chromatography results and structure of TriFs (C_35_H_42_O_20_, >98.6% purity) are shown in Figure 1B,C.

To examine the effects on cell viability of TriFs in pre-osteoblasts, TriFs were treated with 1–100 μM concentration for 24 h, and cell viability was detected using the 3-[4,5-dimethylthiazol-2-yl]-2,5-diphenyltetrazolium bromide (MTT) assay, which is widely used to measure cellular metabolic activity as an indicator of cell viability, cell growth, and cell toxicity. The results revealed that 1 μM TriFs did not affect the metabolic activity, whereas treatment with 5−100 μM TriFs significantly increased the metabolic activity (Figure 1D). We further validated the effects on cell viability of TriFs using BrdU incorporation assay and found that TriFs did not significantly induce BrdU incorporation under the same condition in pre-osteoblasts (Supplementary Figure S2). Thus, TriFs was investigated at low concentrations (1–10 μM) that were determined to be noncytotoxic.

### 2.2. TriFs Accelerates Osteoblast Differentiation and Maturation

**Figure 2:**
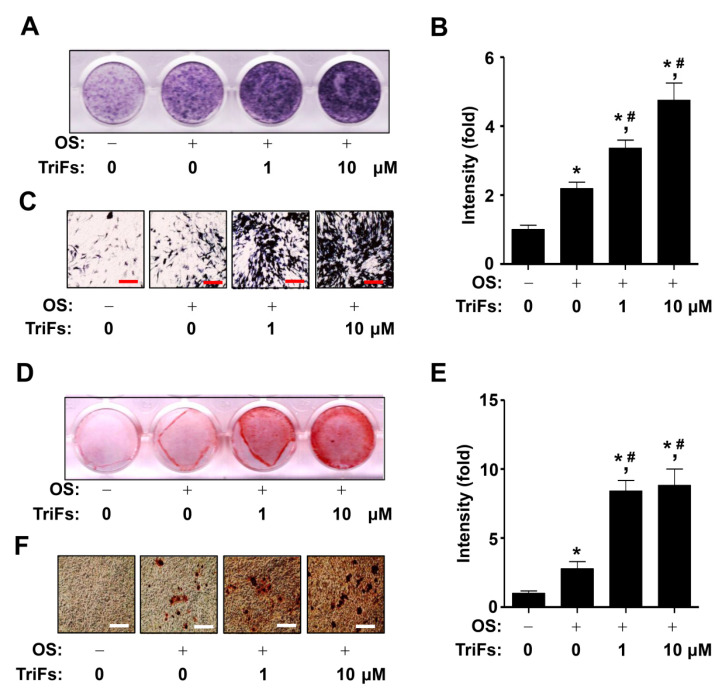
Osteogenic effects of trifloroside (TriFs) on osteoblast differentiation and maturation. (**A**–**C**) Alkaline phosphatase (ALP) staining (**A**) was analyzed after pre-osteoblasts were cultured in osteogenic supplement (OS) medium with TriFs at doses of 0–10 μM for 7 days, and the values (fold) are displayed as a bar graph (**B**). ALP-expressing osteoblasts were observed using light microscopy (**C**). (**D**–**F**) At 21 days, osteoblast maturation was analyzed using an Alizarin red S (ARS) staining assay (**D**), and the values (fold) are displayed as a bar graph. Mineral apposition was visualized using light microscopy (**D**). *, *p* < 0.05 indicates significant differences when compared to the control. #, *p* < 0.05 indicates significant differences when compared to OS. Data are presented as mean ± standard error of the mean (S.E.M.). Data shown are from three independent trials. Scale bar: 50 μm.

Next, to investigate the osteogenic effect of TriFs, differentiation was induced for 7 days in osteogenic supplement (OS) medium with 1–10 μM TriFs, and the osteogenic effect was analyzed using alkaline phosphatase (ALP) staining. Each well of the ALP staining experiments showed that compared to OS alone, 1–10 μM TriFs increased the levels of total ALP (Figure 2A,B). The individual levels of ALP-expressing osteoblasts were visualized under a light microscope, and the osteogenic effects of TriFs were validated (Figure 2C)**.** To examine whether TriFs promotes mineral apposition via osteoblast maturation in pre-osteoblasts, differentiation was induced for 21 days in OS with 1–10 μM TriFs. Alizarin red S (ARS) staining experiments were performed, and each well showed that compared to OS alone, 1–10 μM TriFs increased total ARS staining levels (Figure 2D,E). Visualization of mineralization validated that TriFs promoted mineral apposition by inducing osteoblast maturation (Figure 2F).

### 2.3. TriFs Regulates Multiple Signaling Pathways in Osteoblast Differentiation

**Figure 3:**
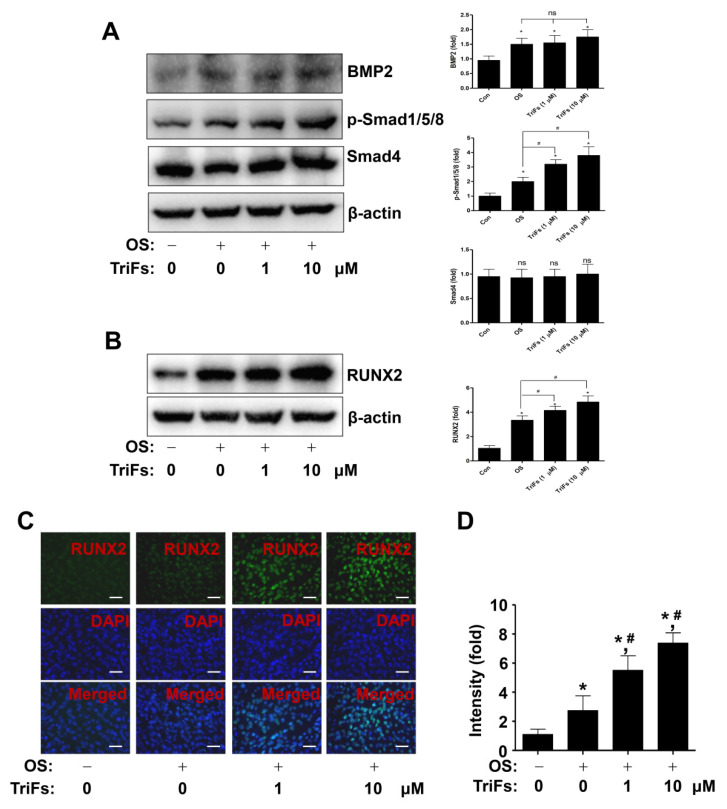
Osteogenic effects of trifloroside (TriFs) on BMP2 signaling molecules and RUNX2 in the nucleus. (**A**,**B**) BMP2, p-Smad1/5/8, Smad4 (**A**), and RUNX2 (**B**) were all probed in equal amounts using Western blotting. The values (fold) were normalized to β-actin and were displayed as a bar graph. (**C**,**D**) RUNX2 (green) and a nuclear marker DAPI (blue) were used to determine nuclear RUNX2 expression and accumulation, which was examined using fluorescence microscopy. The values (fold) are displayed as a bar graph (**D**). *, *p* < 0.05 indicates significant differences when compared to the control. #, *p* < 0.05 indicates significant differences when compared to OS. Data are presented as mean ± standard error of the mean (S.E.M.). ‘ns’, not significant. Data shown are from three independent trials. Scale bar: 50 μm.

To investigate the osteogenic mechanism of TriFs, BMP2 signaling molecules were analyzed using Western blotting. Compared with OS alone, TriFs significantly enhanced the phosphorylation of Smad1/5/8 and the expression of Smad4, but not that of BMP2 (Figure 3A). Trifloroside also increased the expression of BMP2 signaling target protein, the master transcription factor of RUNX2 (Figure 3B). This was further validated using an immunofluorescence assay, which revealed that TriFs treatment increased RUNX2 levels in the nucleus (Figure 3C,D).

**Figure 4:**
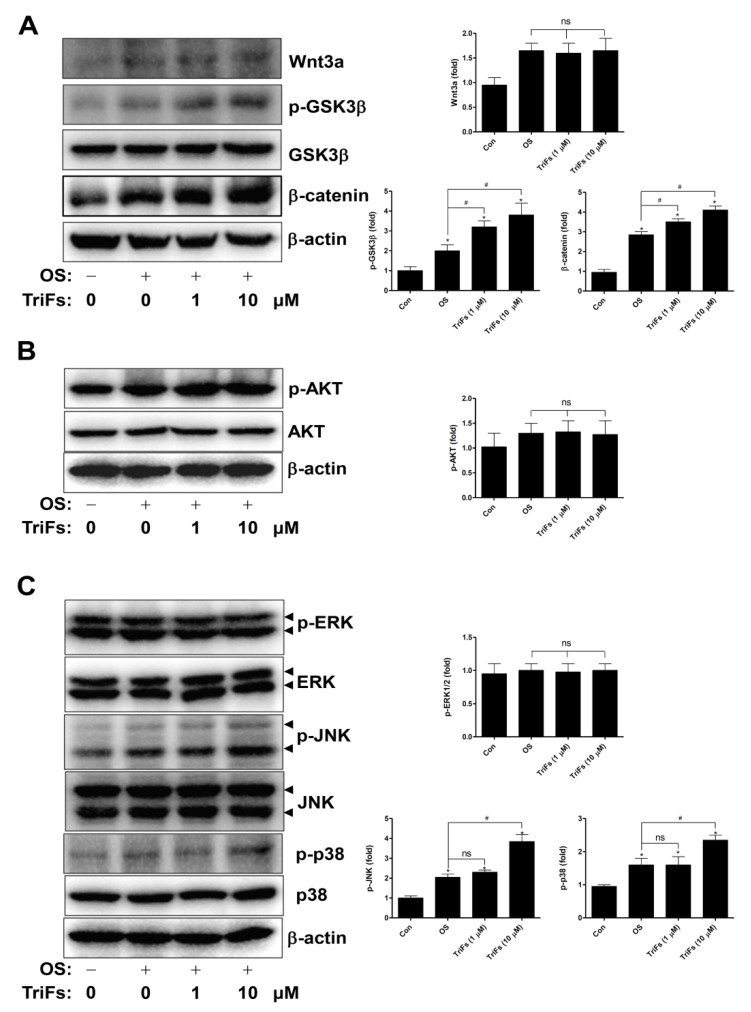
Osteogenic effects of trifloroside (TriFs) on Wnt3a molecules, AKT, and MAPKs. (**A**) Wnt3a, p-GSK3β, and β-catenin were all probed in equal amounts using Western blotting. Data obtained for the lysates were standardized using β-actin on the same sample. (**B**,**C**) AKT, p-AKT (**B**), p-ERK, ERK, p-JNK, JNK, p-p38, and p38 (**C**) were all probed in equal amounts using Western blotting. The values (fold) were normalized to β-actin and were displayed as a bar graph. *, *p* < 0.05 indicates significant differences when compared to the control. #, *p* < 0.05 indicates significant differences when compared to OS. Data are presented as mean ± standard error of the mean (S.E.M.). ‘ns’, not significant. Data shown are from three independent trials.

Next, we analyzed additional signaling molecules involved in RUNX2 expression and activity. The results showed that compared to OS alone, TriFs enhanced GSK3β phosphorylation and β-catenin expression, whereas TriFs did not affect Wnt3a (Figure 4A). Moreover, compared to OS alone, TriFs had no effect on AKT and ERK phosphorylation, whereas TriFs increased JNK and p38 phosphorylation (Figure 4B,C).

### 2.4. TriFs Accelerates Migration and Adhesion during Osteoblast Differentiation

**Figure 5:**
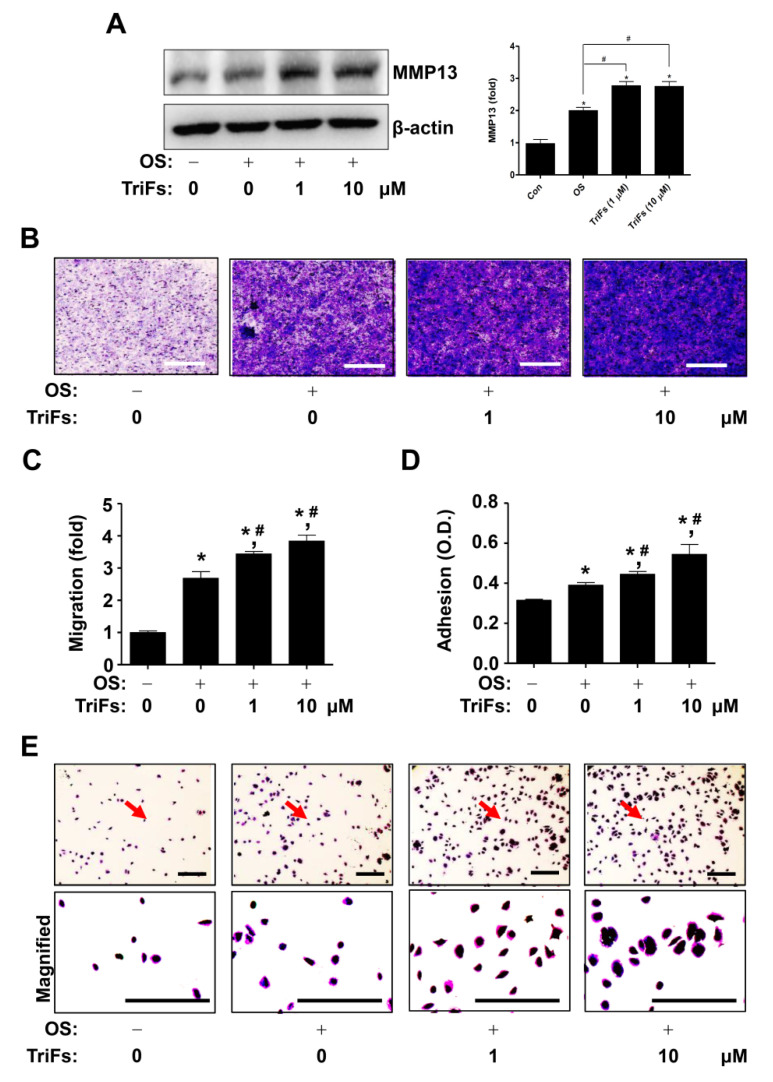
Osteogenic effects of trifloroside (TriFs) on MMP13 production, cell migration, and cell adhesion. (**A**) Western blotting was used to determine MMP13 levels. The values (fold) were normalized to β-actin and were displayed as a bar graph. (**B**,**C**) A Boyden chamber was used to determine the migration of TriFs-treated osteoblasts; a light microscope was used to visualize cell migration (**B**) and the values (fold) are displayed as a bar graph (**C**). (**D**,**E**) An adhesion assay was used to determine the adhesion of TriFs-treated osteoblasts and the numeric values (O.D.) are displayed as a bar graph (**D**). A light microscope was used to visualize cell adhesion, and the red arrows point to the magnified areas (**E**). *, *p* < 0.05 indicates significant differences when compared to the control. #, *p* < 0.05 indicates significant differences when compared to OS. Data are presented as mean ± standard error of the mean (S.E.M.). Data shown are from three independent trials. Scale bar: 50 μm.

The migration and adhesion phenotypes are closely related to osteoblast-mediated bone formation and regeneration. Next, we demonstrated that TriFs regulated cell migration and adhesion during osteoblast differentiation. First, TriF treatment induced the expression of matrix metalloproteinase 13 (MMP13), which plays a critical role in the degradation of extracellular matrix (ECM) and is essential for bone formation and repair (Figure 5A). Second, compared to OS alone, TriFs significantly facilitated penetration across the Matrigel-coated polycarbonate filter in the Boyden chamber, (Figure 5B,C). Third, compared to OS alone, TriFs significantly promoted cell adhesion and morphological stabilization during osteoblast differentiation on Matrigel-coated culture plates (Figure 5D).

### 2.5. Effects of TriFs on Autophagy and Necroptosis in Osteoblast Differentiation

**Figure 6:**
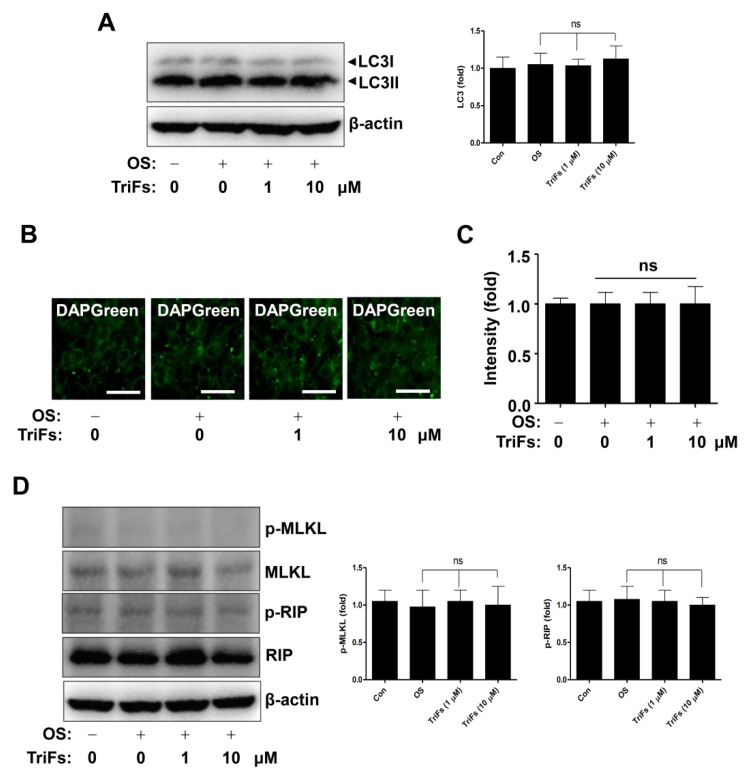
Osteogenic effects of trifloroside (TriFs) on autophagy and necroptosis. (**A**) Western blotting was used to analyze the levels of LC3I and LC3II. The values (fold) were normalized to β-actin and were displayed as a bar graph (**A**). (**B**,**C**) DAPGreen autophagy detection assay was used to determine autophagosome formation (**B**) and the values (fold) are displayed as a bar graph (**C**). (**D**) Western blotting was used to analyze the levels of p-MLKL, MLKL, p-RIP, RIP, and β-actin. The values (fold) were normalized to β-actin and were displayed as a bar graph. Data are presented as mean ± standard error of the mean (S.E.M.). Data shown are from three independent trials. ‘ns’, not significant. Scale bar: 50 μm.

Finally, we investigated whether TriFs regulate autophagosome formation during osteoblastic differentiation. As microtubule-associated protein light chain 3 (LC3) is a key autophagy marker, the expression of LC3 and the conversion of LC3I to LC3II were detected to analyze autophagy using Western blotting. The results showed that TriFs had no appreciable effect on the autophagy markers (Figure 6A). The DAPGreen assay revealed that TriFs did not form autophagic vacuoles (Figure 6B,C). Furthermore, necroptotic regulatory proteins were detected using Western blotting, and the results showed that TriFs had no discernible effect on receptor-interacting serine/threonine-protein kinase (RIP) and mixed lineage kinase domain-like pseudokinase (MLKL) (Figure 6D), indicating that autophagy and necroptosis were not associated with the osteogenic effects of TriFs. Overall, these data suggest that TriFs exerts beneficial effects on osteoblast differentiation and maturation.

## 3. Discussion

Various plant-derived compounds have been reported to promote osteogenesis, osteoid formation, hydroxyapatite synthesis, and mineral apposition via multiple signaling pathways and transcription factors [5,6,15,16]. All of these studies provided novel drug research, development, and clinical applications for treating bone diseases [15,16,17,18,19,20,21]. The present study is the first to investigate the osteogenic effects of TriFs isolated from Gentianae Scabrae Radix on osteoblast differentiation, maturation, and function via intracellular signals and the RUNX2 transcription factor without noticeable autophagic flux and necroptosis.

Osteoblast-mediated bone formation is induced by complex processes via migration and adhesion to bone formation, remodeling, and repair sites; their subsequent differentiation; and osteoblast maturation, leading to osteoid formation and mineral apposition [22,23]. Thus, damage to complex processes causes bone loss in bone diseases such as osteoporosis and periodontitis [7,8]. As is well established, the ALP (a key osteoblast differentiation marker) enzyme is required for the formation of hydroxyapatite crystals through the hydrolysis of organic phosphomonoesters and inorganic pyrophosphate [5,6,24,25]. Herein, we demonstrated that TriFs increases ALP levels during osteoblast differentiation and induces osteoblast maturation, leading to mineral apposition. Alkaline phosphate enzyme activity-mediated hydroxyapatite synthesis is an important process in mineral apposition [24,25]. Alkaline phosphate-deficient mice display bone phenotypes including deformities, fractures, and abnormal mineralization [25]. Therefore, these findings suggest that TriFs exerts anabolic osteogenic effects by accelerating differentiation and maturation.

BMP2 signaling molecules are known to control osteoblast differentiation [26]. BMP2 interacts with BMP receptors and induces signal transduction through Smad1/5/8 phosphorylation to form a Smad1/5/8 and Smad4 complex, leading to nuclear translocation and *RUNX2* expression [27]. Wnt3a signaling is also involved in osteoblast differentiation [28]. Wnt3a interacts with Frizzled and LRP5/6 receptors and increases GSK3β phosphorylation to stabilize β-catenin. Stabilization induces nuclear accumulation and leads to the *RUNX2* expression [29,30]. Thus, RUNX2 expression integrates BMP2 and Wnt3a signaling. In the present study, we demonstrated that TriFs activates Smad1/5/8, inhibits GSK3β, and stabilizes β-catenin, leading to an increase in RUNX2 expression and nuclear accumulation during osteoblast differentiation. MAPKs and AKT signaling proteins are also known to regulate RUNX2 expression and transcriptional activity [2,3,31]. The present study demonstrated that TriFs activates JNK and p38 but not ERK and AKT. RUNX2 is a core transcription factor for osteoblast differentiation and maturation that controls gene expression, especially that of *ALP* [31,32,33]. Given the critical role of RUNX2 in ALP expression, differentiation, and maturation, the present data suggest that TriFs exerts anabolic effects by regulating RUNX2 through Smad1/5/8, β-catenin, JNK, and p38.

Osteoblasts produce MMPs, which are involved in migration, adhesion, and ECM degradation [34,35]. MMP13 is produced in osteoblasts and chondrocytes in bone development and the adult bone and is also considered to play a critical role in bone remodeling and repair [36,37,38]. RUNX2 has been reported to induce MMP13 expression in various cells including pre-osteoblasts [38,39]. In the present study, we demonstrated that TriFs promotes MMP13 production in osteoblasts, migration across the ECM, and adhesion of osteoblasts to the ECM. The migration and adhesion to bone formation, remodeling, and repair niches are required for differentiation and maturation [22,23]. Therefore, our findings suggest that TriFs exerts osteogenic effects by inducing osteoblast migration, adhesion, and subsequent differentiation.

Autophagy and necroptosis are both involved in osteoblast differentiation and maturation. Osteoblast-specific autophagy-deficient mice and osteoblasts were reported to have decreased bone mass and mineral apposition [40]. Kaempferol and Vitamin K2 are reported to promote osteoblast differentiation and maturation by inducing autophagy [41,42]. Autophagy-deficient osteoblasts elevate reactive oxygen species, which increases necroptosis in osteoblasts, and tumor necrosis factor-alpha and chronic ethanol consumption promote necroptotic signaling, resulting in reduced osteogenesis and skeletal formation [40]. In the present study, we explored the possible involvement of TriFs in autophagy and necroptosis in osteoblasts and discovered that TriFs is not linked to autophagic flux and necroptotic signaling in osteoblasts. Thus, our findings suggest that Trifs, regardless of autophagy or necroptosis, promotes osteoblast development and maturation.

## 4. Conclusions

We demonstrated that TriFs enhances osteoblast migration and adhesion, and as a result, stimulates osteoblast differentiation via osteogenic signaling pathways and RUNX2 expression. Our data provide novel evidence that TriFs may be useful as a bone therapy medication that govern osteoblast differentiation and maturation.

## 5. Materials and Methods

### 5.1. Experimental Procedures of Plant Material

Column chromatography was performed using silica gel (70–230 mesh; Merck, Darmstadt, Germany). High-performance liquid chromatography was performed using the Agilent 1260 series system (Agilent Technologies, Santa Clara, CA, USA) with a C18 column (Phenomenex Synergi 10 μ Hydro-RP 80A, 10 μm, 4.6 mm × 250 mm, Torrance, CA, USA). Gentianae Scabrae Radix roots were purchased from a commercial herbal medicine market. A voucher specimen, P542, was deposited in the Natural Products Bank at the National Institute for Korean Medicine Development (NIKOM).

### 5.2. Pre-Osteoblast MC3T3E-1 Cells and Osteoblast Differentiation

Pre-osteoblast MC3T3E-1 cells (Subclone 4, #CRL-2593; American Type Culture Collection, Manassas, VA, USA) were cultivated in α-minimum essential medium without L-ascorbic acid supplemented with 10% fetal bovine serum and 1X Gibco^®^ Antibiotic-Antimycotic (Thermo Fisher Scientific, Waltham, MA, USA) at 37 °C in a humidified environment of 5% CO_2_ and 95% air. The MC3T3E-1 cells were differentiated into osteoblasts by switching to OS medium containing 50 μg/mL L-ascorbic acid and 10 mM β-glycerophosphate. During the differentiation period, the OS was replaced every 2 days. Dimethyl sulfoxide (100%) was used to prepare a 1000× TriFs stock solution, and the vehicle control had a final concentration of 0.1% dimethyl sulfoxide.

### 5.3. Cell Viability

As previously described [43], the MTT assay was used to determine cell viability. Briefly, cells were treated with MTT solution, incubated for 2 h, and then formazan was solubilized with 100% dimethyl sulfoxide. A Multiskan GO Microplate Spectrophotometer (Thermo Fisher Scientific) was used to measure the absorbance at 540 nm.

BrdU incorporation assay was performed using BrdU Cell Proliferation Assay Kit (Biovision, Milpitas, CA, USA) as previously described [44].

### 5.4. ALP and ARS Staining

Osteoblast differentiation was induced for 7 days, and ALP staining was performed as previously described [45]. For the ALP staining assay, the cells were treated with ALP reaction solution (Takara Bio Inc., Tokyo, Japan) for 1 h at 37 °C, and the level of ALP staining was measured using a scanner and light microscope. Differentiation was induced for 21 days and ARS staining was performed as previously described [45]. Cells were stained for 15 min with 2% ARS (pH 4.2) (Sigma-Aldrich, St. Louis, MO, USA), and the ARS staining levels were viewed using a scanner and light microscope.

### 5.5. Western Blotting

Osteogenic, autophagic, and necroptotic-related proteins, as well as their phosphorylation status, were evaluated using Western blotting as previously described [46,47]. The Bradford reagent was used to determine protein concentrations (Bio-Rad, Hercules, CA, USA). Sodium dodecyl sulfate-polyacrylamide gel electrophoresis, polyvinylidene fluoride membranes (Millipore, Bedford, MA, USA), 1× TBS containing 0.05% Tween 20 (TBST), and 5% skim milk were used to examine equal amounts of lysates (20 μg). Specific primary antibodies were incubated overnight at 4 °C, followed by 2 h at room temperature with horseradish peroxidase-conjugated secondary antibodies (1:10,000; Jackson ImmunoResearch, West Grove, PA, USA). Protein signals were detected using the ProteinSimple detection system (ProteinSimple Inc., Santa Clara, CA, USA).

### 5.6. Immunofluorescence

Immunofluorescence assays were performed as previously described [45]. Anti-RUNX2 antibody (1:200; Cell Signaling Technology, Beverly, MA, USA) was incubated overnight at 4 °C, followed by 2 h at room temperature with Alexa-Fluor 488-conjugated secondary antibodies (1:500; Invitrogen, Carlsbad, CA, USA). Nuclei were stained for 10 min at room temperature with a DAPI solution (Sigma-Aldrich). Fluoromount™ Aqueous Mounting Medium (Sigma-Aldrich) was used to mount 8-well chamber slides (Thermo Fisher Scientific) and signal intensity was detected using a fluorescence microscope.

### 5.7. Migration Assay

The migration assay was performed in a Boyden chamber. Briefly, the cells were cultured in a Boyden chamber with Matrigel-coated nuclear pore filters (Corning Life Sciences, Tewksbury, MA, USA). A light microscope was used to track migration. Four regions were randomly selected and quantified.

### 5.8. Cell Adhesion Assay

The cell adhesion assay was performed as previously described [47]. The cells were seeded onto Matrigel-coated 96-well culture plates (Corning Life Sciences), and adherent cells were fixed with 10% formalin and stained with crystal violet for 10 min. A light microscope was used to monitor adhesion.

### 5.9. DAPGreen Autophagy Detection Assay

As previously described [48], Autophagosome formation was monitored using the DAPGreen Autophagy Detection Kit (Dojindo, Japan). Briefly, cells were treated with TriFs after incubation with 0.1 μM DAPGreen solution and rinsed with culture medium. Fluoromount^TM^ Aqueous Mounting Medium (Sigma-Aldrich) was used to mount 8-well chamber slides (Thermo Fisher Scientific), and autophagosomes were viewed under a fluorescent microscope or an intravital multiphoton microscope (SP8-MP) at Gwangju Center, Korea Basic Science Institute (KBSI).

### 5.10. Statistical Analysis

Statistical significance was determined using the Student’s unpaired *t*-test using Prism Version 5 (GraphPad Software Inc., San Diego, CA, USA). Statistical significance was set at *p* < 0.05. Data are presented as mean ± standard error of the mean (S.E.M.).

## Data Availability

The data that support the findings of this study are available from thecorresponding author upon reasonable request.

## Supplementary Materials

The following supporting information can be downloaded at: https://www.mdpi.com/article/10.3390/cells11233887/s1. Supplementary Figure S1. NMR spectra of TriFs from Gentianae Scabrae Radix roots. (A, B) C NMR spectrum (A) and H NMR spectrum (B). Supplementary Figure S2. Effects of TriFs on cell viability in pre-osteoblasts. Cell viability was detected by BrdU incorporation assay. Data shown are from three independent trials.

## Abbreviations

ALP	Alkaline phosphatase.
ARS	Alizarin Red S.
BMP2	Bone morphogenetic protein 2.
β-GP	β-glycerophosphate.
L-AA	L-ascorbic acid.
LC3	Microtubule associated protein light chain 3.
MAPKs	Mitogen-activated protein kinases.
MLKL	Mixed lineage kinase domain-like pseudokinase.
MMP	Matrix metalloproteinase.
MSCs	Mesenchymal stem cells.
MTT	3-[4,5-dimethylthiazol-2-yl]-2,5-diphenyltetrazolium bromide.
OS	Osteogenic supplement medium.
RIP	Receptor-interacting serine/threonine-protein kinas.
RUNX2	Runt-related transcription factor 2.
TriFs	Trifloroside.
