# Supplementary material for: Trifloroside Induces Bioactive Effects on Differentiation, Adhesion, Migration, and Mineralization in Pre-Osteoblast MC3T3E-1 Cells
Source: Cells. 2022 Dec 1;11(23):3887. doi: 10.3390/cells11233887 (PMC9738977; doi:10.3390/cells11233887)

Supplementary Figure Legend

- **Supplementary Figure S1.** NMR spectra of TriFs from *Gentianae Scabrae Radix* roots **. (A, B)** ^{13}C NMR spectrum (A) and ^{1}H NMR spectrum (B)

- **Supplementary Figure S2.** Effects of TriFs on cell viability in pre-osteoblasts**.** Cell viability was detected by BrdU incorporation assay. Data shown are from three independent trials.

Supplementary Fig. S1

A

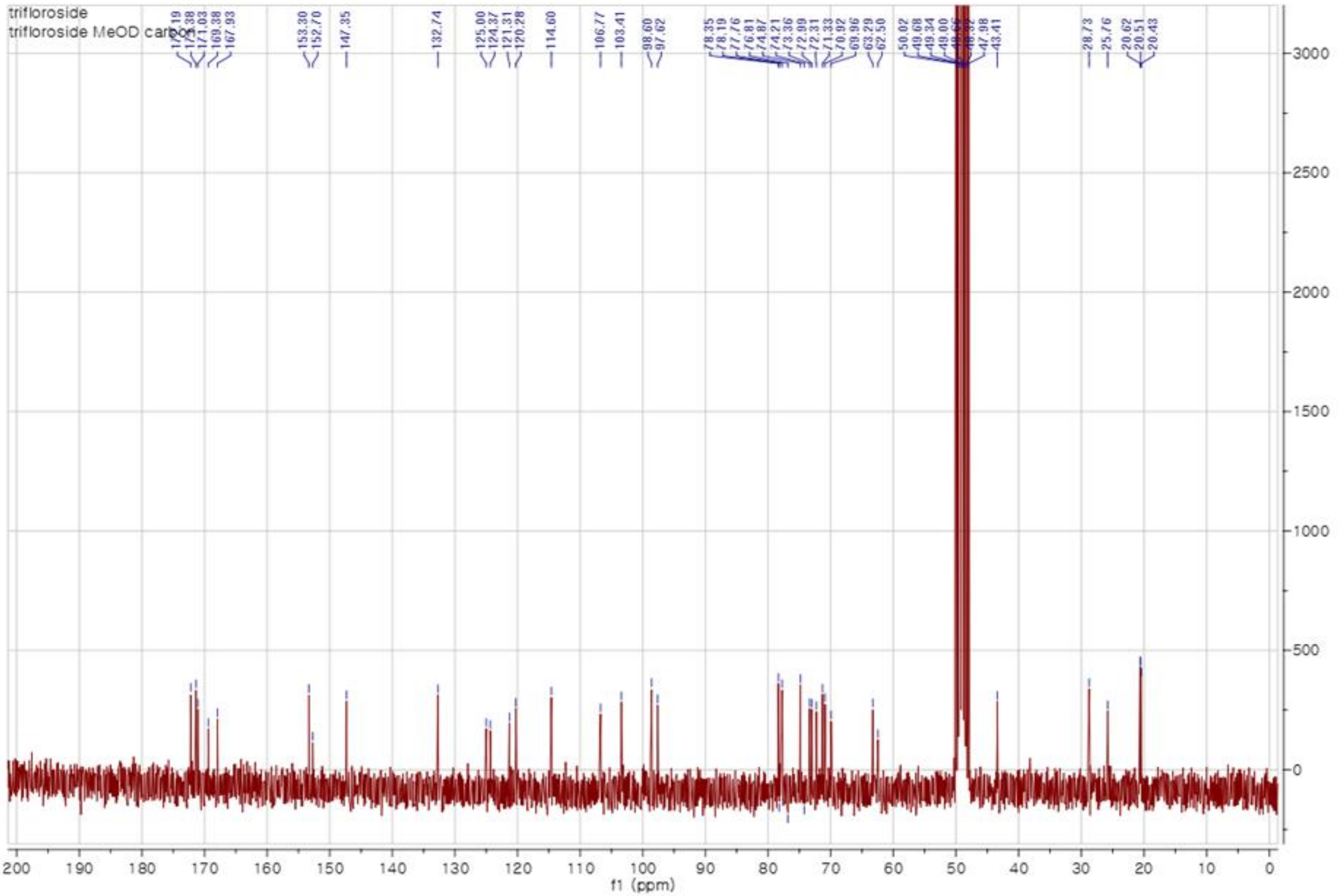

B

Supplementary Fig. S1

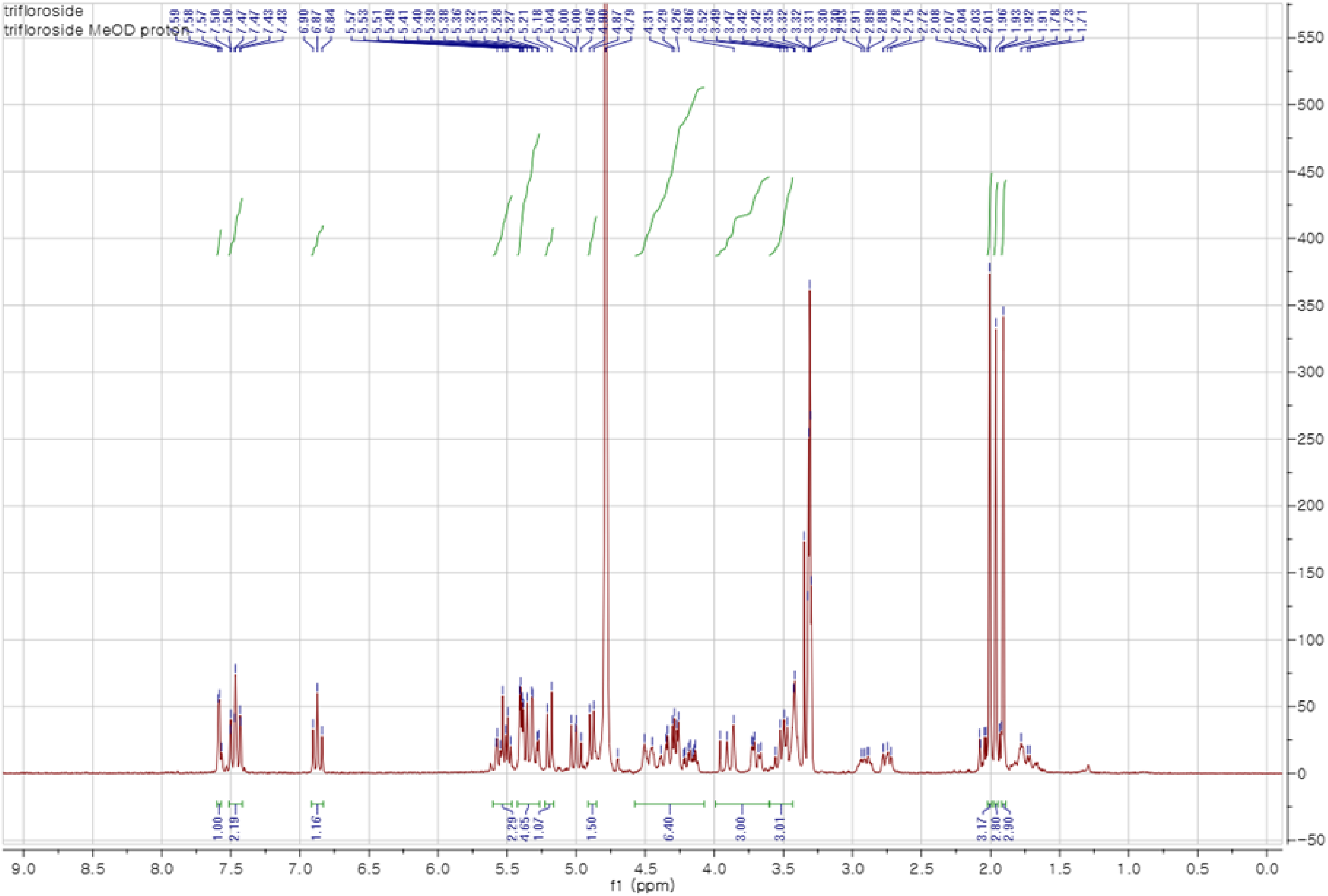

Supplementary Fig. S2

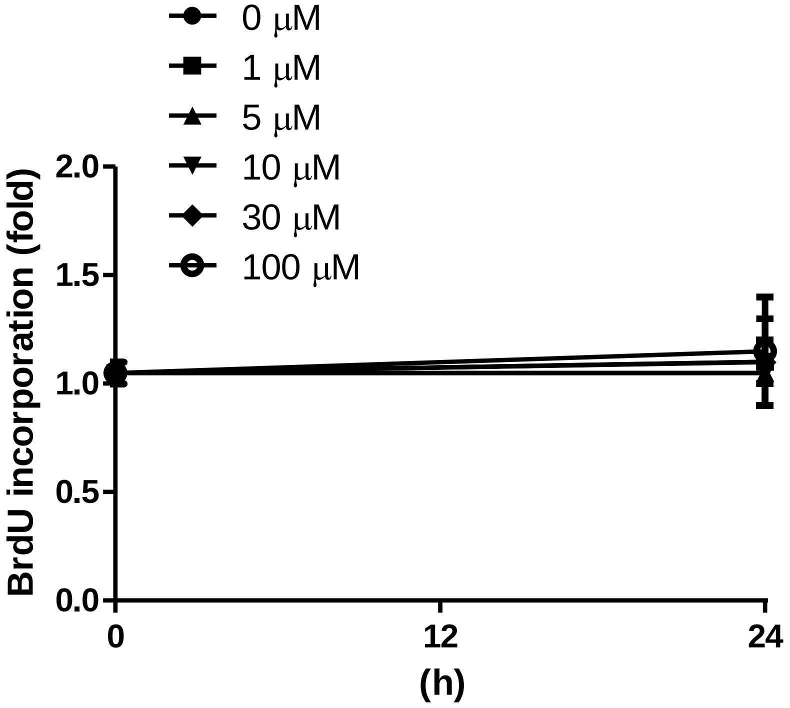

Supplement: Supplementary file 1 [file cells-11-03887-s001.zip › cells-1955378-supplementary.pdf]
